# Acute and subacute cardiovascular effects of synthetic cannabinoid JWH-018 in rat

**DOI:** 10.1007/s11419-025-00720-9

**Published:** 2025-04-16

**Authors:** Onural Ozhan, Necip Ermis, Osman Celbis, Emine Samdanci, Semih Petekkaya, Mucahit Oruc, Ozcan Soylu, Pelin Koparir, Ahmet Acet, Hakan Parlakpinar

**Affiliations:** 1 Department of Pharmacology, Faculty of Medicine, Inonu University, 44280 Malatya, Türkiye; 2 Department of Cardiology, Faculty of Medicine, Inonu University, Malatya, Türkiye; 3 Department of Forensic Medicine, Faculty of Medicine, Inonu University, Malatya, Türkiye; 4 Department of Pathology, Faculty of Medicine, Inonu University, Malatya, Türkiye; 5 Department of Forensic Medicine, Faculty of Medicine, Canakkale Onsekiz Mart University, Canakkale, Türkiye; 6 Department of Chemistry, Forensic Medicine Institute, Malatya, Türkiye

**Keywords:** Synthetic cannabinoids, JWH-018, Heart, Rat

## Abstract

**Purpose:**

This study investigates the cardiovascular effects of the synthetic cannabinoid naphthalene-1-yl-(1-pentylindole-3-yl)methanone (JWH-018) in rats. The research aims to evaluate the pharmacologic, cardiologic, biochemical, and histopathological effects of acute and subacute administration at low and high doses. The primary research question is how JWH-018 impacts heart function, blood pressure, ECG patterns, and cardiac tissue integrity.

**Methods:**

Wistar albino rats were divided into five groups: control, acute low-dose (ALD, 0.5 mg/kg), acute high-dose (AHD, 5 mg/kg), subacute low-dose (SALD, 0.5 mg/kg for 14 days), and subacute high-dose (SAHD, 5 mg/kg for 14 days). Cardiovascular effects were assessed using echocardiography, hemodynamic and ECG analysis, histopathology, biochemical markers, and LC–MS/MS quantification of JWH-018 and its metabolites in heart tissue.

**Results:**

Acute high-dose JWH-018 caused bradycardia and hypotension, while subacute high-dose increased heart rate but continued to lower blood pressure. JWH-018 induced cardiac arrhythmias, conduction blocks, and ischemic ECG changes, with prolonged QT intervals in subacute high-dose rats. Histopathological findings revealed myocardial infarction-like features, including contraction bands and ischemic damage, particularly in subacute groups. Elevated pro-BNP and triglycerides indicated cardiac stress and metabolic effects. JWH-018 and its metabolites were detected in heart tissue, primarily in high-dose groups.

**Conclusions:**

JWH-018 has significant cardiovascular risks, causing heart rate dysregulation, hypotension, arrhythmias, and ischemic damage. These effects depend on dose and duration. The study highlights the potential dangers of synthetic cannabinoids, emphasizing that they should not be considered safe alternatives to natural cannabis.

## Introduction

Synthetic cannabinoids (SCs) are called ‘Spice’ in Europe and ‘K2’ in the USA. The abuse of about 400 chemicals, defined as SCs, is increasing rapidly all over the world. Although generally smoked, vaporization, oral or rectal use has been reported [1]. Several research have looked into using the endocannabinoid system (ECS) to treat myocardial and cerebral ischemia, hypertension, circulatory shock, atherosclerosis, metabolic syndrome, stroke, arrhythmia, and myocardial infarction, among the other vascular diseases [2–6]. Furthermore, studies have shown that cannabis has both beneficial and negative effects when used to treat cardiac problems, and that there is a complex interaction between the ECS, the cardiovascular system, and the immune system [7].

For more than 5 decades, researchers have hypothesized that excessive use of SCs products has resulted in negative cardiovascular consequences. However, the reported frequency of cardiovascular-related problems has steadily increased during the previous decade. Young people are the most common cannabinoid users who develop these issues, and they have no underlying or hereditary relationship to cardiovascular disease. The severity of cardiovascular complications associated with SCs ranges from minor to severe, depending on the kind of issue. Cardiovascular problems have been documented in a number of ways [8]. It is stated that SCs can cause death by creating vasospasm, plaque rupture, thrombus aggregation or myocardial oxygen delivery imbalance, myocardial ischemia and rhythm disturbance. The most common effects of the cardiovascular system due to the use of SCs are tachycardia and increased blood pressure (BP) [9]. Cases with myocardial infarction and QT prolongation have also been reported [10–12].

It is well known that SCs interact with cannabinoid-1 (CB1) and cannabinoid-2 (CB2) receptors in body cells to mimic the effect of *Δ*9-tetrahydrocannabinol (THC), which is the major active ingredient in cannabis or marijuana [13]. The number of new SC derivatives is increasing day after day. The presence of cannabinoid receptors has been demonstrated in the cardiovascular system, myocardial tissues, vascular endothelium, smooth muscle cells, and circulating blood cells [14–17]. CB1 receptors have also been shown in the peripheral nervous system, including the vagus nerve, and can modulate cardiovascular function [18]. Among the reasons for its popularity, today are the sales strategy under the name of legal marijuana, its easy accessibility, its cheap decency, and its negative results in tests developed for THC [10]. During the Covid-19 pandemic, concerns about SCs use have grown [19]. According to preceding reports, SCs intoxication instances had been related to chest pain, angina, arrhythmias, thrombus withinside the coronary artery, acute myocardial infarction, and minor strokes [20]. Studies in which the mechanisms of these effects are being investigated are quite limited.

JWH-018 is the most frequently used SC with a full agonist effect on both CB1 and CB2 receptors, with a short duration of action. While THC compound shows the partial agonistic effect on the CB1 receptor, naphthalene-1-yl-(1-pentylindole-3-yl)methanone (JWH-018) shows full and potent agonistic effect [21, 22]. Acute cannabis exposure is well recognized to cause tachycardia; however, the effect on BP is less reliable. Chronic exposure, on the other hand, has been linked to bradycardia and a drop in BP. When the CB1 receptor is activated in cardiac tissue, it causes a negative inotropic response in the heart. Although CB2 receptor expression has been found in cardiac myocytes, endothelial cells, and smooth muscle cells of coronary arteries, its function is less well understood and requires further research [23].

This study aimed to investigate the effects of the JWH-018 compound, the prototype of SCs, on the cardiovascular system in low dose (LD), and high dose (HD), acute (A) and subacute (SA) time-dependent manner. In this experimental study, JWH-018-related cardiovascular system changes were examined.

## Materials and methods

### Materials

In this study, we used JWH-018 (CAS No: 209414-07-3; Lipomed, Switzerland) compound with molecular formula C_24_H_23_NO. It was stored at + 4 °C until JWH-018 was used. JWH-018 and its metabolites were acquired from Lipomed AG Switzerland. Ketamine hydrochloride (Ketasol %10; Richter Pharma AG, Wels, Australia) and xylazine (Xylazinbio %2; Bioveta Ivanovice na Hané, Czech Republic) were purchased from Biotek Animal Health Products, Malatya, Türkiye. Primary rabbit‐polyclonal caspase‐3 antibody (Neomarker; Lab Vision Corp., Thermo Scientific, Fremont, CA, Cambridge, UK) and mouse monoclonal desmin antibody (Santa Cruz Biotechnology, CA, USA) were used.

### Study design

JWH-018 administered 0.5 mg/kg or 5 mg/kg because Banister and colleagues reported that JWH-018 caused hypothermia and deceleration of heart rate between 0.3 and 10 mg/kg in rats [24]. The study protocol was approved by Inonu University Faculty of Medicine Experimental Animals Ethics Committee with the decision of the ethics committee numbered 2015/A-24. A scientific study permit (No: 21589509/279) was obtained from the Ministry of Justice on March 04, 2015. In the experiments, fifty *Wistar albino* rats weighing 343–429 g were obtained from the Inonu University Experimental Animal Research Center. Rats were maintained in standard cages (12 h of daylight, 12 h of darkness, in ventilated (humidity 60 ± 5%), constant temperature (21 ± 2 °C) rooms) and in special cages. Eight millimeter standard rat pellet diet was used in the feeding process. Randomization was utilized to allocate animals to different rat groups, collect and process data, and analyze the results with investigators who were blind to the treatment groups. This animal experimental study was designed to ARRIVE guidelines [25]. Specific humane endpoints likely included monitoring for significant distress, severe weight loss, and physiological parameters such as HR and BP abnormalities. To eliminate bias in the way the experiment was conducted, a simple randomization procedure was utilized to assign the rats to the groups. Fifty rats were randomly divided into five groups (*n* = 10 for each group) as follows:

1. Control group (C_1–10_): rats were treated with vehicle solution (2 ml of saline containing 2.5% tween 80 and 5% ethanol) intraperitoneally (i.p.) for 14 days.
2. Acute low-dose group (ALD_1–10_): rats were treated with 0.5 mg/kg JWH-018 i.p. for 1 day.
3. Acute high-dose group (AHD_1–10_): rats were treated with 5 mg/kg JWH-018 i.p. for 1 day.
4. Subacute low-dose group (SALD_1–10_): rats were treated with 0.5 mg/kg JWH-018 i.p. for 14 days.
5. Subacute high-dose group (SAHD_1–10_): rats were treated with 5 mg/kg JWH-018 i.p. for 14 days.

### Echocardiographic analysis

Echocardiography (ECHO) was performed blindly under ketamine hydrochloride and xylazine (75 and 5 mg/kg, respectively, i.p.) anesthesia in all groups immediately after the last JWH-018 injection. After the anterior chest wall was shaved in all animals, a transthoracic ECHO was done in a supine position by a researcher (cardiologist, N.E.) who was blinded to the experimental groups. Using a 10-MHz linear transducer probe (GE 10 s parallel Design Inc. Phoenix, USA) and a commercially available ECHO system (Vivid 3; GE Healthcare, Phoenix, USA), standard two-dimensional (2D) and M-mode long- and short-axis (at the midpapillary level) imagines were recorded. Heart rate (HR), left ventricular fractional shortening (LVFS), left ventricular ejection fraction (LVEF), early diastolic filling signal (E), atrial contraction signal (A), mitral valve E/A ratio, and E-wave deceleration time (EDT) were measured and compared among the groups by N.E.

### Hemodynamic and electrophysiological analysis

Hemodynamic data were recorded with the Biopac MP-100 Data Acquisition system (Biopac Systems, Inc., Santa Barbara, CA) after the ECHO analysis. Mean BP was recorded by cannulating the left carotid artery. During this period, rectal temperature controls were performed at 15-min intervals to keep the body temperature of the rats in the range of 36–37 °C. In cases where body temperatures drop, the body blanket, which is laid under the rats, is run and body temperature is balanced. Three-lead electrocardiographic (ECG) electrodes were also used to record ECG changes. In the JWH-018 administered groups, electrophysiological characteristics and pathologic changes in the ECG trace included ST depression, T-wave negative, heart block, cardiac arrhythmia, and QT prolongation. Arrhythmia variety as well as QT times were calculated using Lambeth Convention criteria once the computer data were completed [26]. Blood samples from the inferior vena cava and cardiac tissues were obtained once the test regimen was completed. Since hemodynamic records were taken for 2 h, blood and heart tissue samples were taken by euthanasia of 4 rats every day. The serums were separated by centrifugation from the blood taken into the gel biochemistry tube. Heart tissue and serum samples were stored in a deep freezer at − 80 degrees until biochemical analyzes began.

### Histopathological analysis

Heart tissue samples were preserved in 10% formaldehyde at the end of the experiment. After routine tissue follow-up, the paraffin-embedded samples were cut in 4µ thickness and stained with Hematoxylin and Eosin (H&E). Staining was performed in a Ventana Benchmark XT immunohistochemical staining device (Ventana Medical Systems, Roche Group, Tucson, AZ, USA) to evaluate desmin and caspase-3 antibodies in sections. All staining was evaluated under the light microscope. Semi-quantitative scoring was done between 0 and 3 for the intensities of desmin staining. Scoring system: 0: no staining, 1: mild staining, 2: moderate staining, 3: severe staining [27].

### Biochemical analysis

The animals’ blood samples were centrifuged for 10 min at 2000 rpm. The serum samples were placed in tubes and stored in the freezer (at a temperature of − 80 °C). Frozen materials were transported to a + 4 °C unit one day before biochemical analysis to dissolve. Then, troponin-I, myoglobin, pro-brain natriuretic peptide (pro-BNP), low-density lipoprotein (LDL), high-density lipoprotein (HDL), triglyceride, and total cholesterol parameters were analyzed.

### Quantification of JWH-018 and its metabolites by liquid chromatography tandem mass spectrometry in the heart tissue

Liquid chromatography tandem mass spectrometry (LC–MS/MS) method for the identification and quantification of JWH-018 and JWH-018’s metabolites in rat heart tissue was developed. The method was validated in rat heart homogenates and was significantly sensitive to quantitate the concentrations of JWH-018 and JWH-018’s metabolites. The method determined by Poklis et al. was used in the preparation of heart tissue samples [28, 29].

#### Heart tissue calibration method

Low quantity limit 10 ng/g, medium quantity limit of 80 ng/g and high quantity limit of 100 ng/g to JWH-018 metabolites in cardiac tissue and calibration samples of JWH-018-free (1:4 tissue:water) were prepared using rat heart tissue homogenates. Tissues taken from rats were homogenized with a glass homogenizer and diluted ¼ percent with pure water. The samples were kept overnight after being vortexed and added while being vortexed drop by drop with 2 ml of cold acetonitrile on the following day. Then, the samples were centrifuged at 3500 rpm for 10 min and kept at − 40 °C for at least 2 h. The upper part containing acetonitrile was taken with a disposable pipette and transferred to a clean tube. The extracts were then dried using Teknosem TAB-40-WEL Evaporator. The residue was dissolved in 100 µl mobile phase. The samples were taken into insert vials and made ready for analysis. A seven to eightfold difference between the peak areas of JWH-018 and its metabolites, and the metabolites that are difficult to separate from each other, were properly separated by solid-phase extraction method.

#### Solid-phase extraction

The heart tissue samples were prepared using OASIS HLB cartridges. The cartridges were conditioned with ethyl acetate, methanol and water, respectively. The tissue sample was weighed and diluted with water in a ratio of 1: 4, and then homogenized for 1 min at 4400 rpm. On the homogenate, 5 ml of water was added and after centrifugation at 4400 rpm for 10 min, the upper clear part was placed in the conditioned cartridge. It was washed with 5% methanol solution by volume. It was dried under high vacuum for at least 30 min. The elution step was completed twice with 0.5 ml methanol and 0.5 ml ethyl acetate. It was centrifuged at 14,000 rpm for 10 min and placed in 0.2 ml LC–MS/MS inserts. JWH-018 and its *N*-(2-hydroxypentyl), *N*-(3-hydroxypentyl), *N*-(4-hydroxypentyl), *N*-(5-hydroxypentyl) and *N*-pentanoic acid metabolites were quantificated in the heart tissue by LCMS-8040 (Shimadzu Scientific Instruments, Columbia, MD).

### Statistical analysis

For detecting even minor effects, the required sample sizes used in this experiment were identified using statistical power analysis. The sample sizes necessary for a power of 0.80 were estimated using NCSS software. The Kolmogorov–Smirnov test was used to determine if the data conformed to a normal distribution. While Kruskal–Wallis H test and Mann–Whitney *U* test are used in some analysis of data (ECHO and biochemical analysis in serum), Tukey’s test was applied after the intergroup ANOVA, since the other data analyses (the animal weight, mean BP, ECG measurements and LC–MS/MS analysis) conformed to normal distribution. Test results after normal distribution are presented as mean ± standard deviation (SD). Non-parametric test results are presented as median (min–max). Pearson Chi-square test with exact approach was used to analyze categorical variables. A *p* value less than 0.05 is statistically significant. IBM Statistical Package for the Social Sciences version 24.0 for Windows was used for analysis.

## Results

One animal died in the ALD and SAHD groups during hemodynamic measurements.

### The body weight of animals

The body weight of rats treated with SA low and high dosage of JWH-018 decreased significantly at the end of the experiment. The results were presented in Table 1.

**Table 1:** Body weight of animals

Groups (*n* = 10)	The beginning of the experiment (g)	The end of the experiment (g)
Control	390 ± 39	390 ± 39
ALD	381 ± 38	381 ± 38
AHD	387 ± 24	387 ± 24
SALD	386 ± 35	346 ± 31^ac^
SAHD	382 ± 22	325 ± 20^abc^

The values were given as mean ± SD

^a^Significant differences compared to the Control group (*p* < 0.05)

^b^Significant differences compared to the ALD group (*p* < 0.05)

^c^Significant differences compared to the AHD group (*p* < 0.05)

### Hemodynamic analysis

The results are shown in Table 2. Briefly, mean BP values indicated a significant decrease in the acute and subacute high-dose JWH-018-treated rats when compared with the control and ALD groups (*p* < 0.05). When compared to the other groups, SAHD JWH-018 treatment induces a prolonging in the interval of QT durations (*p* < 0.05).

**Table 2:** Hemodynamic analysis

Groups (*n* = 10)	Mean BP (mm-Hg)	QT interval (ms)	ST depression	T negativity	Heart block	Cardiac arrhythmia
Control	71.4 ± 1.6	137 ± 38	0	0	0	0
ALD	70.7 ± 0.8	133 ± 16	4	1	6	5
AHD	57.0 ± 1.3^ab^	133 ± 6	1	1	6	3
SALD	67.6 ± 1.7	159 ± 25	1	1	4	3
SAHD	64.7 ± 1.8^ab^	192 ± 29^abcd^	3	0	4	1

The values were given as mean ± SD

^a^Significant differences compared to the Control group (*p* < 0.05)

^b^Significant differences compared to the ALD group (*p* < 0.05)

^c^Significant differences compared to the AHD group (*p* < 0.05)

^d^Significant differences compared to the SALD group (*p* < 0.05)

**Fig. 1:**
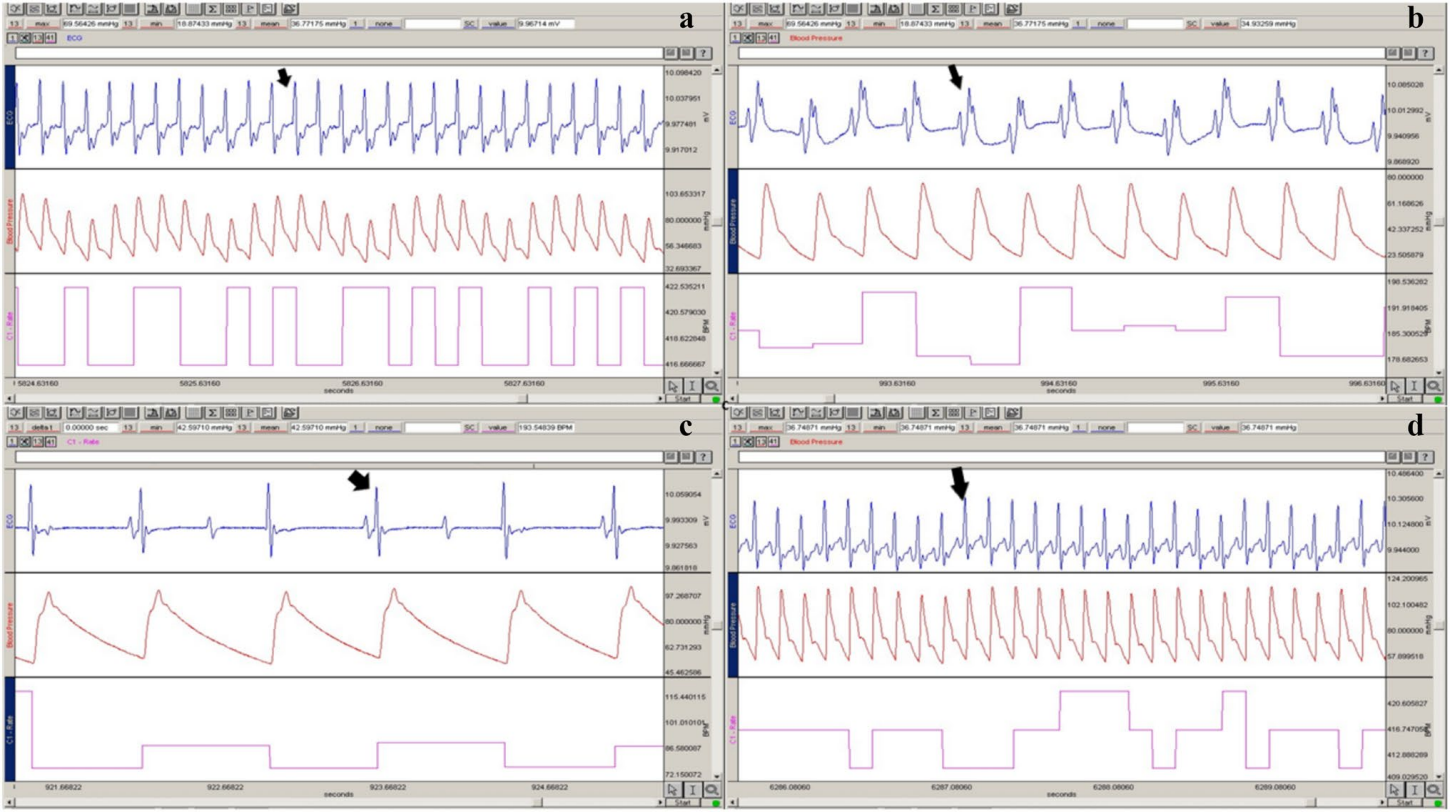
The following are examples of ECG (blue), BP (red), and HR (pink) signals obtained using the BIOPAC MP100 data acquisition system: Branch block (**a**), ST depression (**b**), atrioventricular block (**c**) and T negativity (**d**) in the ALD group

The normal ECG pattern was seen in the control group rat’s ECG data. In the JWH-018-treated groups, considerable abnormalities in the ECG trace, such as ST depression, T negativity, heart block, and cardiac arrhythmia were found (Fig. 1). T negativity was not observed only in the SAHD group. According to the results of the analyses made with the group variable and ST depression, T negativity, heart block, and cardiac arrhythmia variables, no statistically significant relationship was found (*p* > 0.05) (Table 2).

### Echocardiographic examinations

**Fig. 2:**
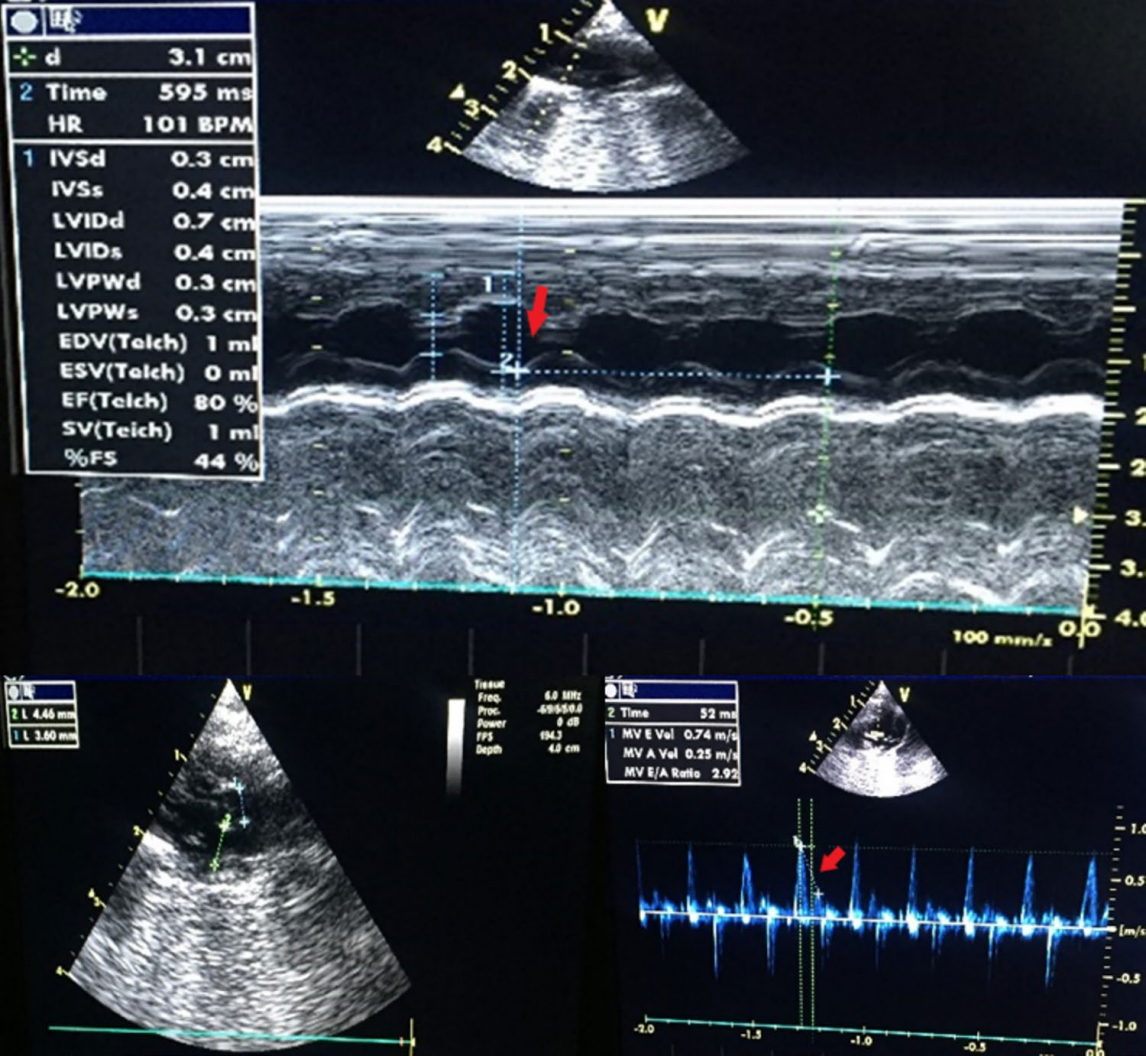
M-mode (top) and two-dimensional (left bottom) echocardiographic images and Doppler tissue images (right bottom) of the left ventricle in Control group. *IVSd* interventricular septal end diastole, *IVSs* interventricular septal end systole, *LVIDd* left ventricular internal diameter end diastole, *LVIDs* left ventricular internal diameter end systole, *LVPWd* left ventricular posterior wall thickness in diastole, *LVPWs* left ventricular posterior wall thickness in systole, *EDV* end-diastolic volume, *ESV* end-systolic volume, *EF* ejection fraction, *SV* stroke volume, *FS* fractional shortening, *MV E Vol* mitral valve flow E-wave, *MV A Vol* mitral valve flow A-wave

The effects of JWH-018 on the variables of ECHO are presented in Fig. 2 and Table 3. In brief, HR values indicated a significant increase in the SALD group when compared with the control and acute JWH-018-treated groups, whereas this parameter was found as a significant increase in the SAHD group when compared to the control and AHD groups (*p* < 0.05). However, JWH-018 administration did not alter LVEF, LVFS, E-wave, A-wave, E/A ratio and EDT variables among the groups (*p* > 0.05).

**Table 3:** Echocardiographic examination findings

Groups (*n* = 10)	HR (beats/min)	LVEF (%)	LVFS (%)	E-wave (m/s)	A-wave (m/s)	E/A	EDT (ms)
Control	235 (101–377)	75 (68–88)	39 (33–53)	0.62 (0.51–0.90)	0.27 (0.12–0.59)	2.26 (1.02–6.60)	33 (26–52)
ALD	271 (126–406)	82 (74–87)	45 (38–51)	0.55 (0.46–0.76)	0.27 (0.12–0.56)	2.93 (1.01–5.16)	41 (22–59)
AHD	178 (113–361)	80 (68–91)	44 (33–57)	0.77 (0.57–0.94)	0.21 (0.10–0.41)	3.76 (1.42–5.70)	41 (30–48)
SALD	396^abc^ (290–464)	81 (55–88)	42 (26–53)	0.67 (0.55–0.74)	0.27 (0.15–0.40)	2.56 (1.58–4.72)	37 (22–44)
SAHD	331^ac^ (250–427)	84 (65–89)	52 (31–54)	0.55 (0.44–0.86)	0.24 (0.13–0.45)	3.15 (1.18–4.27)	44 (30–63)
*p* value	**0.001**	0.755	0.599	0.115	0.444	0.430	0.336

Bold values denote statistical significance at the *p* < 0.05 level

The values were given as median (min–max)

*HR* heart rate, *LVEF* left ventricular ejection fraction, *LVFS* left ventricular fractional shortening, *E* early diastolic filling signal, *A* atrial contraction signal, *E/A* peak velocity of E-wave/peak velocity of A-wave, *EDT* E-wave deceleration time

^a^Significant differences compared to the Control group (*p* < 0.05)

^b^Significant differences compared to the ALD group (*p* < 0.05)

^c^Significant differences compared to the AHD group (*p* < 0.05)

### Histopathological examinations

**Fig. 3:**
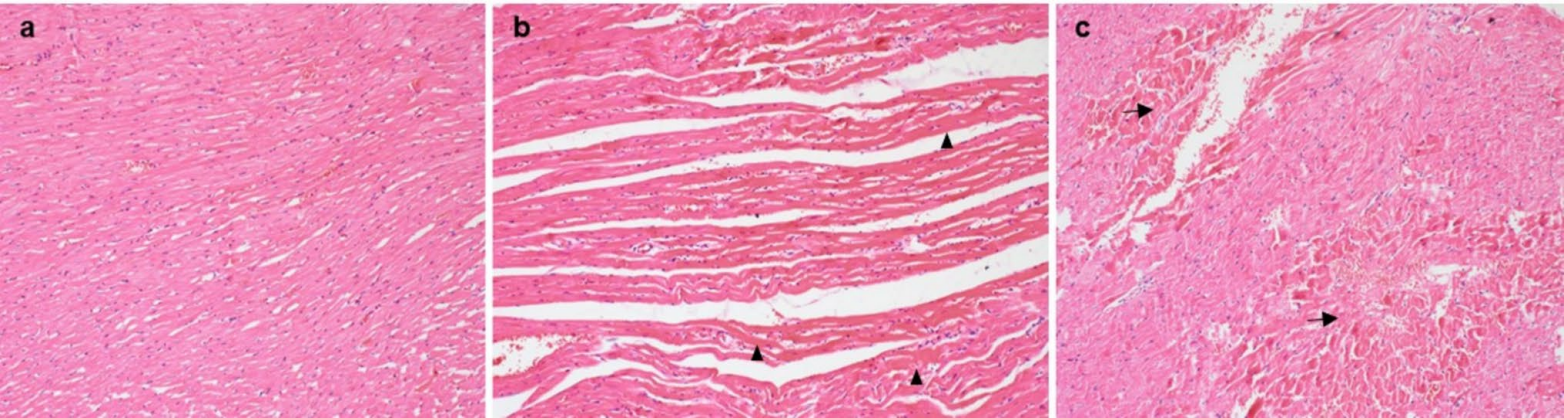
**a** Normal heart histology. H&E × 100, **b** Wavy appearance of myocytes and increased eosinophilia in SALD and SAHD groups (arrowhead). H&E × 100, **c** SALD and SAHD groups, areas of karyolysis compatible with ischemic findings (arrow). H&E × 100

In the H&E sections, there was normal heart histology in the control group (Fig. 3a). In the hearts of all rats in the other groups, contraction bands with necrosis in focal areas, karyolysis characterized by the absence of nuclei in myocytes, were interpreted as focal and histopathological changes consistent with early stage myocardial infarction associated with ischemia. The SALD and SAHD groups had more common and significant signs of ischemic changes compared to the other groups. These changes were observed to be compatible with 4–12 h of ischemia. The wavy appearance in myocytes was such that their cytoplasm contained eosinophilia and contraction band (Fig. 3b and c).

**Fig. 4:**
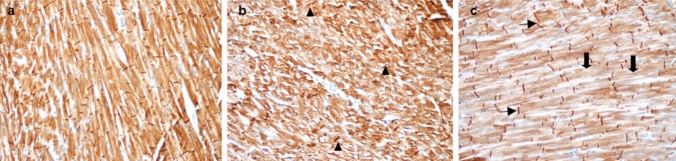
**a** Normal staining in myocytes with desmin antibodies, Control group, Score 0. × 200, **b** Slightly decreased staining and moderately increased contraction bands in myocytes with desmin antibodies (arrowhead), SAHD group, Score 2. × 200, **c** Decreased staining (thick arrow) and increased contraction bands (thin arrow) in myocytes with desmin antibodies, SALD group, Score 3. × 200

Desmin expression in groups was demonstrated in brown with 3,3ʹ-diaminobenzidine (DAB) chromogen using immunohistochemistry method. When the severity and prevalence of staining with desmin antibody in the groups were evaluated, weak, moderate and intense staining patterns were observed. It was noted that the control group showed a moderate and homogeneous staining pattern in terms of staining intensity and prevalence. This pattern was consistent with the staining pattern seen in myocytes in normal heart tissue. In SALD and SAHD group cases in which contraction bands were prominent, desmin accumulation was observed to be intense, especially in myocyte firming aggregates. In the cases of these two groups, it was noted that desmin stained weakly in terms of staining intensity and intensity in the ischemia areas seen as small foci (Fig. 4b and c). Staining of SALD and SAHD group cases with desmin antibody was consistent with histomorphologically observed early signs of acute myocardial infraction and ischemia findings.

**Fig. 5:**
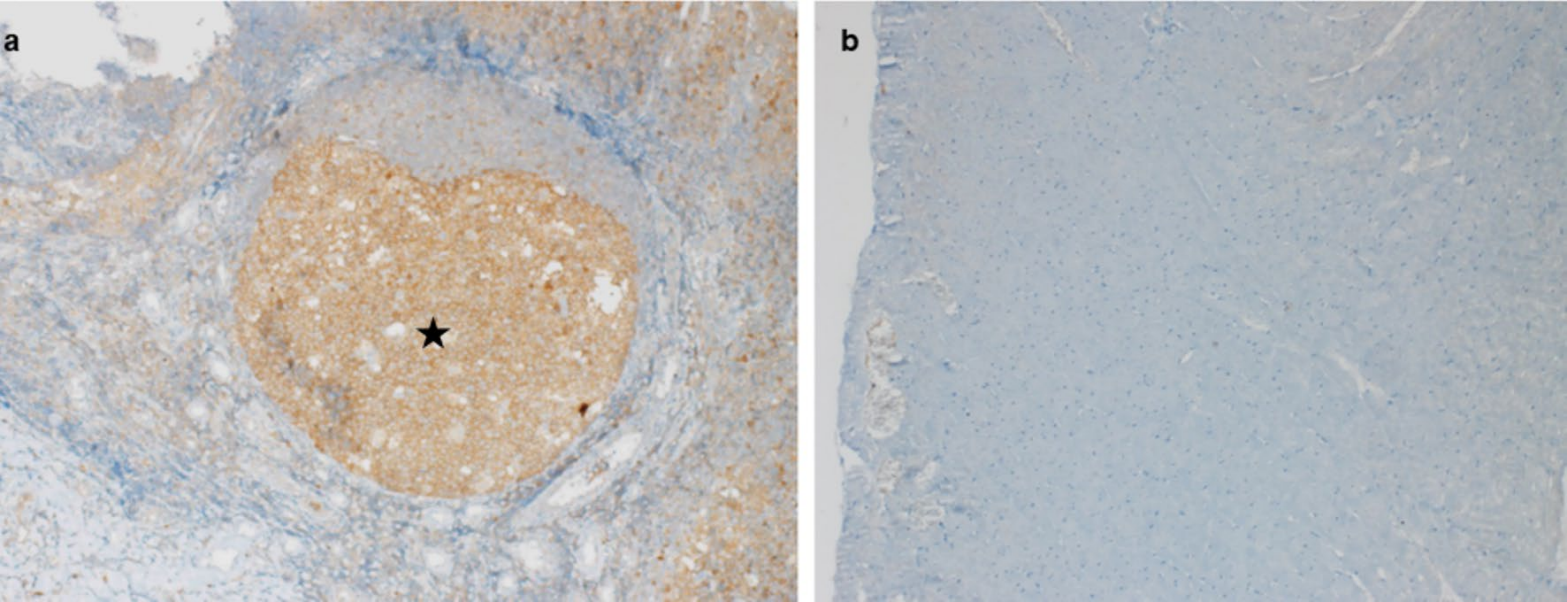
**a** Caspase-3 antibody positive control, follicle germinal center, tonsil (star). × 100, **b** Negative staining of the heart with caspase-3 antibody, SAHD group. × 100

Tonsillar tissue was used as a positive control in the same glass and it was observed that the caspase-3 antibody worked in this positive control (Fig. 5a). Heart tissues in all groups were stained negatively with caspase-3 antibody.

### Biochemical analysis

The serum biochemical parameters are summarized in Table 4. In brief, when the groups were compared in terms of the troponin-I, myoglobin, pro-BNP, LDL, HDL, triglyceride (TG) and total cholesterol variables. pro-BNP and triglyceride variables were statistically significant (*p* < 0.05).

**Table 4:** Serum biochemical parameters at the end of the experiment

Groups (*n* = 10)	Troponin-I (pg/ml)	Myoglobin (ng/ml)	pro-BNP (pg/ml)	LDL (mg/dl)	HDL (mg/dl)	TG (mg/dl)	Total cholesterol (mg/dl)
Control	13,741 (8856–39,474)	713 (205–1178)	20 (20–20)	7.2 (3.4–21.4)	29 (24–44)	57 (38–77)	45 (40–81)
ALD	25,050 (3008–50000)	856 (205–1200)	20 (20–52)	12.8 (2.9–22.3)	29 (20–37)	49 (35–121)	54 (47–62)
AHD	32,048 (5767–50,000)	1088 (356–1200)	20 (20–52)	7.8 (5.1–14.9)	30 (23–36)	81 (50–99)	56 (38–59)
SALD	34,337 (13,849–50,000)	978 (319–1200)	20 (20–165)	11.9 (8.6–16.1)	32 (26–37)	92^a^ (68–198)	59 (50–76)
SAHD	26,835 (2163–50,000)	978 (651–1200)	35^a^ (20–165)	11.9 (4.3–15.6)	29 (17–34)	115^ab^ (39–210)	52 (49–61)
*p*	0.446	0.372	**0.046**	0.555	0.617	**0.020**	0.340

Bold values denote statistical significance at the *p* < 0.05 level

The values were given as median (min–max)

*Pro-BNP* pro-brain natriuretic peptide, *LDL* low-density lipoprotein, *HDL* high-density lipoprotein

^a^ Significant differences compared to the Control group (*p* < 0.05)

^b^ Significant differences compared to the AHD group (*p* < 0.05)

### LC–MS/MS analysis

In the quantification of JWH-018 and its metabolites by LC–MS/MS analysis, JWH-018 and JWH-018 metabolites could not be detected in the control and ALD groups in the heart tissue. JWH-018 and all other JWH-018 metabolites except for JWH-018 *N*-(2-hydroxypentyl) derivative were determined in the AHD group. Levels of JWH-018 and metabolites were found as follows: JWH-018 (6.868 ± 5.117) ng/mg, JWH-018 *N*-(3-hydroxypentyl) (0.103 ± 0.048) ng/mg, JWH-018 *N*-(4-hydroxypentyl) and *N*-(5-hydroxypentyl) (0.129 ± 0.069) ng/mg, JWH-018 N-pentanoic acid) (0.148 ± 0.038) ng/mg, respectively. JWH-018 was determined in SA JWH-018 groups, SALD (0.068 ± 0.0499) ng/mg and SAHD (0.626 ± 0.418) ng/mg, whereas JWH-018 metabolites were not detected; all results are given in Table 5.

**Table 5:** The number and amount of JWH-018 and JWH-018 metabolites determined in the heart tissue

Groups (*n* = 9)	JWH-018 (ng/mg)	JWH-018 N-(2-hydroxypentyl) (ng/mg)	JWH-018 N-(3-hydroxypentyl) (ng/mg)	JWH-018 N-(4-hydroxypentyl) and N-(5-hydroxypentyl) (ng/mg)	JWH-018 N-pentanoic acid) (ng/mg)
Control	ND	ND	ND	ND	ND
ALD	ND	ND	ND	ND	ND
AHD	9/9 (6.868 ± 5.117)^a,b^	ND	4/9 (0.103 ± 0.048)	9/9 (0.129 ± 0.069)	6/9 (0.148 ± 0.038)
SALD	9/9 (0.068 ± 0.0499)^b^	ND	ND	ND	ND
SAHD	9/9 (0.626 ± 0.418)	ND	ND	ND	ND

The values were given as mean ± SD

Quantification limit is 0.01 ng/mg

*ND:* not detected

^a^Significant differences compared to the SALD group (*p* < 0.05)

^b^Significant differences compared to the SAHD group (*p* < 0.05)

## Discussion

Cannabinoid receptors may be found all over the cardiovascular system. The myocardium, human coronary artery, endothelial, and smooth muscle cells, as well as presynaptic sympathetic nerve terminals that innervate the cardiovascular system, all express the CB1 receptor. In addition to the myocardium, CB2 receptors have been discovered in human coronary endothelium and smooth muscle cells. Endocannabinoids are generated in endothelium and smooth muscle cells, as well as heart tissue, and their amounts in the blood may be measured. Despite this, the endocannabinoid system is unlikely to have a significant role in the control of cardiovascular function under normal circumstances [30]. JWH-018 is a SC with full agonist effect on both CB1 and CB2 receptors. It is known that the CB1 receptor affinity of SCs is 100 times higher than THC. Due to far more potent receptor activity, it is quite reasonable to expect the cardiovascular effects to be stronger and more problematic.

The negative effects of SCs on the cardiovascular system have been shown in previous studies [31–33]. According to our analyses of cardiac results, the HR response of JWH-018 was found as different due to dose and duration of application. While single high-dose administration resulted with reduced HR, chronic administration resulted with increased HR irrespective of JWH-018 dose. In the AHD group, reduced HR values were accompanying with reduced mean BP. In long-term, BP drop was continuing despite increased HR, especially in the rats are given prolonged high-dose JWH-018. In the related literature, the most frequently reported cardiovascular effect of SCs is a significant decrease in arterial BP, heart contraction and HR [30, 34, 35]. Despite the fact that numerous studies show that SCs related cardiovascular depression effects are mediated by CB1 receptors, they may also have vascular and cardiac effects that are independent of CB1 and CB2 receptors. The role of CB1 receptors in the vasodepressor response has been demonstrated by the relief of hypotension when a CB1 selective antagonist is used [36]. Hypotension caused by cannabinoid and the complete absence of bradycardia in CB1 receptor-deficient mice are the main evidence showing the effect of CB1 receptors on these cannabinoid-related effects [37]. In anaesthetized hypertensive mice, Batkai and colleagues discovered that CB1 receptor agonists reduce contractility and normalize BP [38]. Recently, it was shown that presynaptic CB1 receptor stimulation inhibits norepinephrine release both in vitro and in vivo [39, 40]. JWH-018 and HU-210 are both SCs that act as agonists at CB1 and CB2 receptors, but their differences in chemical structure, receptor binding affinity, and metabolism lead to distinct pharmacological and toxicological effects. JWH-018 contains an indole core with a nitrogen atom, distinguishing it from THC, which lacks nitrogen. HU-210, in contrast, is a classical cannabinoid structurally related to THC, but is fluorinated and contains a bicyclic structure instead of an indole ring. The presence of nitrogen in JWH-018’s indole ring system significantly alters its binding properties and metabolic stability, leading to more potent and toxic effects compared to THC and HU-210 [22]. Furthermore, the hypotensive response to HU-210, a SC, remained intact when sympathetic tone was reduced by ganglionic blockade and vascular tone was restored by vasopressin infusion, even if the bradycardic effect was gone [41]. Above-mentioned data indicate that cannabinoid-induced bradycardia is caused in the short term by inhibition of sympathetic tone to the heart; however, the hypotensive response is directly related to vasodilation [42]. According to our long-term results, increased HR response can be explained by reflex tachycardia for chronic reduced peripheral resistance and hypotension. Furthermore, cannabinoids have a strong ability to block acetylcholine release from heart.

In the current study, the effect of JWH-018 on cardiac structure and function was evaluated using transthoracic ECHO, although we did not recognize functional and structural changes in terms of ejection fraction or fractional shortening and any of diastolic function parameters. In addition, level of serum pro-BNP which is a sign of impaired cardiac functions was found to be increased in long-term high-dose JWH-018-treated group. It can be accepted that this situation is compatible with the cardio depressant effect of SCs as proven before. Pacher et al. highlighted that the hypotensive action of a SC, HU-210, is predominantly due to a decrease in ventricular contractility in pentobarbital-anesthetized mice in vivo, employing pressure–volume conductance [43]. In accordance with this finding, in another study, Wagner et al. reported decreased cardiac index and resulting BP by same SC using radiolabeled microsphere technique [41].

It has been reported in many clinical cases, SCs can cause cardiac arrhythmias and fatalities. CB1/CB2 effects appear to accumulate over time, altering cardiac function through multiple pathways: chronic CB1 activation leads to progressive downregulation of CB1 receptors, altering receptor sensitivity and cardiac autonomic regulation [53]. CB1-mediated inhibition of norepinephrine release may induce compensatory autonomic changes that contribute to progressive cardiac dysfunction [39]. Chronic CB2 activation has been linked to increased oxidative stress and inflammation in cardiac tissue, leading to electrophysiological changes that may prolong the QT interval [15]. In our preclinic study, cardiac arrhythmia frequency was found to be increased in all JWH-018 groups. It has been noticed that there is QT prolongation in rats in the group treated with SAHD JWH-018, unlike in other groups. Moreover, QT analysis, resembling arrhythmia risk, showed prolongation in long-term cannabinoid use, despite increased HR especially in the high-dose group. Al Kury et al. previously demonstrated that endogenous cannabis can produce arrhythmias in rat ventricular myocytes by blocking the function of voltage-dependent Na^+^ and L-type Ca^2+^ channels in the absence of CB1 and CB2 receptor activation [44]. In another study, Li et al. found that anandamide, an endocannabinoid, reduced L-type Ca^2+^ current in ventricular myocytes and delayed the length of action potential in cardiac tissues via CB1 but not CB2 receptors. Beside this, anandamide facilitated the inactivation of L-type Ca^2+^ current and inhibited its recovery from inactivation [45]. Recently, Yun et al. investigated the effect of JWH-30, a synthetic cannabinoid on duration of action potential and QT interval. They observed that inhibiting the human ether-a-go-go related gene (hERG) channels in rabbit Purkinje fibers shortened the duration of action potential, and that intravenous administration of JWH-030 (0.5 mg/kg) at the ECG measurement in rats lengthened the QT interval [46]. There is mounting evidence that using SCs increases the likelihood of a clinically significant lengthening of the rate-corrected QT interval of the ECG. Torsades de pointes is the main arrhythmia connected to delayed ventricular repolarization and, therefore, QT interval lengthening. This can progress to deadly ventricular fibrillation and is related to cellular origin of early-after depolarizations and enhanced repolarization dispersion. Therefore, QT prolongation caused by both prescription medications and illicit substances has some relevance [47]. In cases where SCs were detected as a result of toxicological studies conducted in the autopsy series, body fluids and tissue samples examined, the causes of death were usually due to cardiac problems. Cardiac problems identified include causes such as myocardial infarction, dilated cardiomyopathy, cardiomegaly, arrhythmias, and decontamination [48–50]. In the present study, during evaluation of QT interval and arrhythmia, we have also evaluated the ischemic ECG changes such as ST segment depression and T-wave negativity in all JWH-018-administered rats. Although we did not detect any ECG changes resembling ischemia, all JWH groups have more ischemic ECG findings despite prior reported coronary vasodilatory effect of cannabinoids [41]. This ECG changes may be result of decreased BP causing reduced coronary perfusion or increased HR causing supply demand mismatch. However, we did not find cardiac troponin-I elevations in serum as a myocardial injury biomarker in contrast to our histopathological observations which showed ischemic circumstance in the cardiac tissue. Histopathologically, we found morphological changes compatible with the first 4–12th h of ischemia in the SALD and SAHD groups. Although these ischemic changes were not seen on the ECG findings, we thought that they reflected the decreased BP result. Histopathological findings related to arrhythmia detected on ECG were not observed in the JWH-018 groups.

One more thing should be emphasized is the metabolic effect of JWH-018. The ECS regulates hunger and energy balance in the central nervous system, principally through managing both the homeostatic and hedonic components of food intake. By activating CB1 receptors in brain areas implicated in energy control, both endogenous and exogenous cannabis can promote food absorption, change the release of orexigenic and anorexic mediators, and boost hedonic valuation (i.e., the hypothalamus and mesocorticolimbic system) [51]. In contrast, agents with specific antagonistic effects for the CB1 receptor have been shown to suppress food intake and reduce body weight in laboratory animals [52]. Contrary to this knowledge, we found that all rats lost weight when long-term JWH-018 was used, regardless of dose. Cooper reported that among the common side effects of SCs of moderate severity, there may be a decrease in body weight due to loss of appetite [12]. Dalton et al. reported that weight loss was linked to a dose-dependent downregulation of CB1 receptors that lasted throughout chronic exposure [53]. We also found that triglyceride levels in blood lipids were decreased, possibly related to weight loss of rats.

Previously, it is well shown that metabolic enzymes are involved in the biotransformation of SCs. The main ring involved in the molecular structure of SCs is metabolized, especially by the CYP1A enzyme [54]. In some studies, CYP2C9 and CYP1A2 enzymes have been responsible for the metabolism of JWH-018 [55]. The elimination half-life of JWH-018 in animals is approximately 2 h, meaning it is largely cleared within 8–10 h [58]. However, some hydroxylated and carboxylated metabolites have longer half-lives and persist in tissues for 24 h or more, depending on metabolic clearance [55]. This suggests that while JWH-018 itself exerts strong immediate effects, its metabolites could contribute to prolonged pharmacological and toxicological consequences, including cardiovascular stress. The persistence of JWH-018 metabolites in the SAHD group despite lower JWH-018 levels suggests that these metabolites might sustain the toxic effects even after the parent compound has been cleared. Given that SC metabolites can have partial or full agonist activity, their continued presence could extend receptor activation, leading to prolonged hypotension, tachycardia, and arrhythmias [57]. At least nine mono-hydroxylated metabolites of JWH-018 have been found. It has been shown in studies that these metabolites bind to the CB1 and CB2 receptors [56, 57]. It was found that the heart value was between 0.16 and 1.63 ng/mg in the rats in the AHD group. In addition, it was estimated that the heart value was between 0.02 and 0.08 ng/mg in the SALD group. It is detected at lower levels than the rats in the ALD group. One of the possible explanations of these results is that it is oxidized by CYP2C9 and CYP1A2 cytochrome P450 isoforms in drug metabolism and it was thought that the increase in metabolites and the storage of substance metabolites in the organs were due to the effect of UGT2B7 found in hepatic tissue and UGT1A3 main function isoforms found in extraheptic tissue in the conjugation step [11]. The HRs of the rats increased statistically significantly in the groups in which JWH-018 was administered subacutely, compared to the groups in which it was administered acutely. This circumstance may lead to increase renal perfusion and increased renal excretion of JWH-018 and its metabolites. JWH-018 tests on animals have indicated a half-life of some 2 h [58, 59]. Considering that it takes approximately 4–5 half-lives for a drug to be completely removed from the body, the levels of the drug and its metabolites may have been low in SA JWH-018 administered groups due to increased renal clearance with increased HR.

In addition, it was determined that the heart value was between 0.02 and 0.08 ng/mg in the SAHD group. Based on this relationship, in a clinical study, two volunteers received 100 and 150 mg SCs containing 2.9% JWH-018. JWH-018 serum concentration peaked 5 min after inhalation, reaching 8.1 mcg/L and 10.2 mcg/L. However, it is reported that the concentration decreases rapidly after 1 h and cannot be detected in the 24th h [60]. Compared to the AHD group, there was a higher blood level and substance-metabolite accumulation in the tissues. The reason for this situation was thought to be the decrease in the metabolic rate of the drug in excess and/or the toxic effect of the drug on the tissues. At lower doses, metabolic enzymes may process the drug more efficiently, preventing significant accumulation in the heart. Increased HR in the SALD group might have further accelerated drug clearance by enhancing renal and hepatic blood flow, leading to faster elimination. Low-dose JWH-018 might preferentially accumulate in the brain or fat tissue rather than the heart, making it undetectable in cardiac tissue samples. Previous studies show that SCs have a high affinity for lipid-rich tissues, which could explain the lack of detection in the heart at low doses [59].

JWH-018 and metabolite determinations are mostly measured in tissues such as serum, blood, oral fluid, urine, brain, kidney, lung, liver and spleen. Although JWH-018 has been determined in heart tissue before, the study was in mice and only the level of JWH-018 was assayed [61]. On the other hand, we aimed to examine the JWH-018 level in the heart tissue along with the other 5 metabolites, and we determined the levels of 4 metabolites that can be separated from each other correctly. In addition, unlike the other study, the fact that we looked at 4 separable metabolites that we reached as consumables, rather than a few metabolites in serum and heart tissue, constitutes another important uniqueness of our study.

## Conclusions

SC JWH-018 beside known psychoactive properties has some detrimental cardiac and vascular effects such as tachycardia, bradycardia, hypotension, arrhythmia and QT prolongation depending on the dose and duration of application. It seems that JWH-018 has dose- and duration-dependent increase or decrease in HR, high-dose-related decrease in BP, cardiac arrhythmia, conduction blocks, and ischemic ECG changes. The information presented in this study suggests that SCs should not be thought of as secure and authorized substitutes for cannabis. Instead, the increased toxicity of SCs may be as a result of the interactions between the many SCs present and their active metabolites that still have a high affinity for CB1 and CB2 receptors, emphasizing the potential risk associated with using these drugs.
